# Management of high-dose olanzapine poisoning in a young child under limited resource setting: a case report from Nepal

**DOI:** 10.1097/MS9.0000000000002652

**Published:** 2024-10-16

**Authors:** Pramodman Singh Yadav, Popular Pokhrel, Sashank Bhattarai, Pratik Adhikari, Abinash Dev, Aishworya Upadhaya, Shipra Chaudhary

**Affiliations:** a B.P. Koirala Institute of Health Sciences; b Department of Pediatrics and Adolescent Medicine, B.P. Koirala Institute of Health Sciences, Dharan, Nepal

**Keywords:** case report, olanzapine, pediatric poisoning, resource-limited settings

## Abstract

**Introduction:**

Olanzapine, an atypical antipsychotic, is widely used for treating psychiatric conditions such as schizophrenia and bipolar disorder. Accidental overdose in children is rare but can lead to severe clinical effects. This case report discusses the management of a 5-year-old male who accidently ingested 180 mg of olanzapine, the highest reported dose in a child around 5 year.

**Case presentation:**

A 5-year-old boy accidentally ingested 180 mg of Olanzapine, resulting in loss of consciousness and central nervous system depression. He exhibited hyperglycemia, elevated lactate, and prolonged prothrombin time, but no significant cardiovascular issues. Following intubation and supportive care, including intravenous medications and mechanical ventilation, the child gradually improved. He was discharged in stable condition with follow-up instructions.

**Discussion:**

Olanzapine toxicity in children presents with a variety of symptoms, including somnolence, hypotension, and neurological impairments, which are dose-dependent. Even in case of an exceptionally high overdose, the absence of cardiovascular toxicity supports safety profile of olanzapine. Common laboratory findings include hyperglycemia, elevated liver enzymes, and metabolic disturbances. Management involves airway protection, supportive care, and monitoring, as no specific antidote exists. Prompt and appropriate care, even in severe cases under limited resource settings, can lead to favorable outcomes.

**Conclusion:**

In cases of high-dose accidental olanzapine poisoning in children, it is essential to begin quick intervention and comprehensive supportive care to achieve successful outcomes and can be managed even in limited resource settings. Preventive measures are crucial to avoid such incidents, and careful monitoring is essential in managing pediatric olanzapine overdose.

## Introduction

Highlights

- The authors present a case of the highest dose of olanzapine poisoning (180 mg) reported so far in the age group of children around 5 years.
- The child’s presentation with hyperglycemia, mild anemia, altered coagulation parameters, and common side effects like CNS depression, miosis, and respiratory compromise, alongside the absence of significant cardiovascular toxicity despite the high dose, underscores the complexity of olanzapine poisoning.
- In a resource-limited setting, effective management is focused on supportive care, including airway protection, continuous monitoring, and early interventions leading to successful outcomes.

Olanzapine is an atypical antipsychotic drug of thienobenzodiazepine group majorly used for the treatment of psychoses, schizophrenia, bipolar disorders and depression along with off-label clinical uses like agitation, delirium, anorexia nervosa, chemotherapy-induced nausea and vomiting (CINV) control in children^1^. Olanzapine overdose usually reflects its known therapeutic actions, which include tachycardia, agitation or aggression, dysarthria, extrapyramidal dystonic effects, sedation or coma, small pupils, blurred vision, respiratory depression and hypotension^2^. Olanzapine overdose in children is uncommon, often associated with more remarkable side effects and requires more potent intervention than in adults^3^. We hereby report a case of massive olanzapine overdose in a 5-year-old where he consumed 12 tablets of 15 mg (180 mg total) and its management in a limited resource setting. This case report has been drafted according to CARE guidelines^4^.

## Case presentation

A 5-year-old male presented with aggressive behavior and loss of consciousness following the accidental ingestion of multiple Olanzapine tablets (12 tablets of 15 mg each). He was born at term via emergency lower (uterine) segment cesarean section (LSCS) for premature rupture of membrane (PROM), with a birth weight of 3.5 kg. The child had cried immediately after birth and had no perinatal complications. He was fully immunized as per the National Immunization Program (NIP) and had typical developmental milestones, recently starting school. The patient’s father had been consuming Olanzapine regularly for the last six months, which was accidently ingested by the child.

Upon physical examination, the patient’s vitals were as follows: pulse 80/min, blood pressure 111/64 mm Hg (50th percentile), respiratory rate 28/min, temperature 97.6°F, and SpO2 99%. The child weighed 18 kg and measured 102 cm in height. The general examination revealed an ill-looking child with pinpoint pupils that were non-reactive to light. The capillary refill time was less than 2 seconds. Chest examination showed bilateral equal air entry with no added sounds. Cardiovascular examination found S1S2 normal with no murmurs. The abdomen was soft, non-distended and non-tender. Central nervous system examination showed a Glasgow Coma Scale (GCS) of 12/15 (E3V4M5), but the initial assessment was limited due to sedation.

Laboratory investigations revealed several notable findings, including hyperglycemia, elevated lactate levels, prolonged prothrombin time (PT) elevated chloride, neutrophilia, lymphopenia, mild anemia, reduced RBC count. Arterial blood gas (ABG) was performed, which revealed a slightly alkaline pH of 7.402, pCO_2_ of 41.0 mmHg, elevated pO_2_ of 114 mmHg and a normal bicarbonate (HCO_3_^−^) level of 25.2 mmol/l, indicating a mixed respiratory and metabolic component. Additionally, the lactate level was elevated at 7.0 mmol/l, suggesting potential tissue hypoxia or metabolic distress and high pO_2_ likely reflects efforts to correct potential tissue hypoxia or metabolic imbalance. Significant laboratory investigations have been tabulated as below (Table 1). Remarkable complete blood count findings revealed total leukocyte count of 10 090 cells/μl, which is within the normal range, with a predominant neutrophil percentage of 86%. The patient also presented with mild anemia, as indicated by hemoglobin 11.5 g/dl and reduced RBC count 3.40 million/μl. All complete blood count (CBC) findings have been tabulated below (Table 2).

**Table 1:** Biochemical and arterial blood gas profile

Parameter	Patient’s value	Reference value
Sodium (Na+)	140.7 mmol/l	135–145 mmol/l
Potassium (K+)	4.1 mmol/l	3.5–5.0 mmol/l
Ionized calcium (Ca++)	1.12 mmol/l	1.12–1.32 mmol/l
Chlorine (Cl−)	114 mmol/l	98–108 mmol/l
HCO3− (actual)	25.2 mmol/l	22–28 mmol/l
HCO3− (standard)	24.0 mmol/l	22–28 mmol/l
Glucose	169 mg/dl	70–140 mg/dl (random)
Lactate	7.0 mmol/l	0.5–2.2 mmol/l
pH	7.402	7.35–7.45
pO2	114 mmHg	80–100 mmHg
pCO2	41.0 mmHg	35–45 mmHg
Total protein	6.9 g/dl	6.0–8.3 g/dl
Albumin	4.3 g/dl	3.5–5.0 g/dl
Total bilirubin	0.6 mg/dl	0.1–1.2 mg/dl
Conjugated bilirubin	0.1 mg/dl	0.0–0.3 mg/dl
ALT (alanine aminotransferase)	11.0 U/l	5–30 U/l
ALP (alkaline phosphatase)	231 U/l	140–320 U/l
AST (aspartate transaminase)	30 U/l	20–60 U/l
PT (prothrombin time)	15 sec	11–13.5 sec
INR (international normalized ratio)	1.07	0.8–1.2

**Table 2:** Complete blood count (CBC) and red cell indices

Parameter	Value	Reference value
Hemoglobin (Hb)	11.5 g/dl	11.5–15.5 g/dl
Packed cell volume (PCV)	32.3%	30–43%
Total leukocyte count	10 090 cells/μl	4000–11 000 cells/μl
Neutrophil	86%	40–70%
Lymphocyte	06%	20–40%
Monocyte	04%	2–10%
Eosinophil	04%	1–6%
Basophil	00%	0–1%
Platelet Count	261 000 cells/μl	150 000-400 000 cells/μl
Red blood cell	3.40 million/μl	4.0–5.5 million/μl
Mean corpuscular volume (MCV)	83.1 fl	77–95 fl
Mean corpuscular hemoglobin (MCH)	28.9 pg	26–34 pg
Mean corpuscular hemoglobin concentration (MCHC)	32.5%	31–37%
Mean platelet volume (MPV)	9.5 fl	7.5–11.5 fl

The patient was admitted and oxygen was administered via a face mask. Initial management included intravenous ranitidine and ondansetron. An Electrocardiogram (ECG) was performed, revealing no significant findings. The patient showed gradual agitation and respiratory rate was increasing after admission and was thus intubated. Intravenous Ceftriaxone was added to the treatment regimen to prevent hospital-acquired pneumonia infection. The patient underwent regular monitoring, and a stat dose of intravenous Mannitol was administered to manage any potential intracranial pressure changes.

The patient was extubated after 15 hours of mechanical ventilation and maintained on oxygen via a face mask. Feeding was initiated and oral medications were started. The patient showed gradual improvement and throughout the hospital stay, the patient was closely monitored for any signs of complications and received supportive care to manage symptoms and prevent adverse effects. By the time of transfer, the patient’s vital signs had stabilized and his clinical condition had improved significantly. The patient was discharged with follow-up instructions for continued observation and safety measures to prevent future incidents.

## Discussion

Olanzapine is a commonly used atypical antipsychotic drug of thienobenzodiazepine group with improved safety profile and effectiveness at the usual therapeutic dosage. The antipsychotic activity of olanzapine is attributed to its dopaminergic antagonistic activity while it’s multi-receptor action that is antagonism to dopaminergic (D1, D2, D4), serotoninergic (5-Hydroxy tryptamine (HT) 2A, 5HT2C), histaminergic (H1), cholinergic (M1, M2, M3, M4, M5) and α1-adrenergic receptors results in variable clinical symptoms during acute poisoning^5^. Frequently observed symptoms in acute olanzapine overdose are lethargy/coma, tachycardia, anticholinergic syndrome, confusion/agitation/delirium, dysarthria, jerks/myoclonus, extrapyramidal effects, hypertension or orthostatic hypotension with unpredictable alterations between CNS depression and agitation together with miosis^6^. While the usual therapeutic dosage range for olanzapine is 5–15 mg/day, toxicity can be observed if consumption exceeds this range. The maximum amount of olanzapine consumed in a child around five years that has been reported in the literature is 75 mg. In our case, the child had consumed 180 mg, which is the highest quantity of olanzapine consumed till date^2,7^.

Bond *et al.* ^8^ reported accidental olanzapine poisoning in a 6-year-old who had consumed 10 mg olanzapine and presented with slurred speech, staggering gait, and extreme drowsiness. Lankheet *et al.* ^9^ reported a 2-year-old female who had ingested 30 mg of olanzapine and presented in a state of sleeping with eyes not opening on stimuli; showed tachycardia, hypertension (later hypotension), miosis with normal light reflexes. Swift *et al.* ^10^ reported a case of olanzapine overdose in a 1.5-year-old with presentations of sinus tachycardia, dry mucous membranes, and hypoactive bowel sounds. Catalano *et al.* ^11^ presented the case of an 18-month-old boy who ingested 30–40 mg of olanzapine, which resulted in significant symptoms, including respiratory distress and mental status changes. Tanoshima *et al.* ^12^ reported a one year old female with a consumption of 20–50 mg of olanzapine and exhibited lethargy, somnolence, fever, jitters, ataxia, tremors. Dokur *et al.* ^13^ reported a case of 3-year-old female having consumed 20 mg olanzapine mimicking opioid toxicity as tachycardia, somnolence with insufficient cooperation, rapid atrial rhythm, hypotension, sinus rhythm, prolonged PR interval, supraventricular premature complexes and T-wave negativities in anterior deviations. Yokoyama *et al.* ^14^ reported an olanzapine overdose of 20 mg in a 2-year-old female with presentation of loss of consciousness, hypotension and constricted pupils with a normal light reflex. Singh *et al.* ^7^ reported a case of 2-year-old male who had consumed 75 mg olanzapine with presentations of somnolence, hypotension, tachycardia and miosis. Kumar *et al.* ^15^ reported two cases of a 2-year-old having consumed 20 mg olanzapine with chief presentation of altered sensorium and abnormal ECG and an 8-year-old having consumed 30 mg with the presentation of excessive drowsiness and normal ECG.

Systematic review of safety of Olanzapine in young children by Flank *et al.* ^16^ reported weight gain in 78%, sedation in 48%, extrapyramidal symptoms in 9%, electrocardiogram abnormalities in 14% and blood glucose abnormalities in 4% and elevation in liver function tests in 7%. A systematic review of cardiovascular effects following antipsychotic medication overdose by Tan *et al.* ^17^ revealed thirteen pediatric patients (<7 years) with olanzapine overdose. CVS abnormalities as tachycardia, hypotension were noted whereas no pediatric case revealed ventricular dysrhythmia or death. This suggests that significant CVS toxicity is not expected with olanzapine poisoning, but some minor abnormality is seen in most of the cases.

From the review of the pediatric cases of accidental olanzapine poisoning, it is evident that children under and around 5 years old who ingest olanzapine often present with a range of symptoms, including somnolence, hypotension, tachycardia, and neurological impairments such as altered sensorium and miosis. The severity of symptoms appears to be dose-dependent, with higher doses leading to more pronounced and potentially life-threatening effects, including respiratory distress and cardiovascular abnormalities. However, even at lower doses, olanzapine poisoning can mimic other toxic syndromes, such as opioid toxicity, complicating the clinical diagnosis and management. In contrast to all the above cases, our patient consumed 180 mg of olanzapine, the highest reported dose in a child around 5 years old. Despite this, the child exhibited no significant cardiovascular toxicity or abnormal ECG findings, which supports the notion that cardiovascular toxicity is more commonly associated with typical antipsychotics than with atypical ones like olanzapine.

Common laboratory abnormalities in olanzapine poisoning are hyperglycemia, hyperprolactinemia, elevated CPK, hypokalemia, hyperbilirubinemia, leukocytosis, hyponatremia, thrombocytopenia, elevated liver enzymes^18^. In our case, the child developed hyperglycemia, possibly due to a combination of stress response and Olanzapine’s known effects on glucose metabolism. Coagulation parameters with slight abnormalities, indicates potential issues with blood clotting, possibly linked to liver function or the pharmacological impact of the drug. In the bloodwork, an increased neutrophil count is suggestive of an acute stress or inflammatory response, while a reduced lymphocyte count might reflect the immunosuppressive effects of Olanzapine. The child also exhibited signs of mild anemia, potentially due to the physiological stress of the overdose or a side effect of the drug. The elevated lactate level in the arterial blood gas analysis, though non-specific, is indicative of a metabolic disturbance, which could be secondary to olanzapine’s impact on mitochondrial function or tissue hypoxia due to sedative effects. These findings are crucial; especially in resource-limited settings where an option for serum monitoring of drug level is not available.

There is no specific antidote for olanzapine poisoning. Immediate management should focus on establishing and maintaining the airway, ensuring adequate oxygenation and ventilation, and considering the potential involvement of multiple drugs. Gastric lavage and activated charcoal administration can be beneficial, especially if done early, as charcoal can significantly reduce olanzapine absorption. Continuous cardiovascular monitoring is essential to detect arrhythmias, and hypotension should be managed with intravenous fluids and sympathomimetic agents, avoiding those with beta-agonist activity^1^.

In our case, the management of the patient during the unstable state following olanzapine ingestion involved a comprehensive treatment approach focused on stabilization. Initial airway management included early intubation due to compromised respiratory function, ensuring adequate ventilation and oxygenation. Continuous monitoring of vital signs was implemented to detect any changes, and intravenous fluid resuscitation was provided to maintain hydration and support blood pressure. Supportive medications, such as ranitidine for gastrointestinal protection and ondansetron for nausea, were administered. A stat dose of mannitol was also given to address potential intracranial pressure, albeit as a precautionary measure. Ceftriaxone was added to the regimen to prevent hospital-acquired pneumonia, a risk associated with the patient’s condition and prolonged hospital stay. To ensure nutritional needs were met, after 24 hours of hospitalization, as the patient’s condition improved, feeding was initiated following extubation, which was done after 15 hours of mechanical ventilation. The multidisciplinary team, which included pediatric intensivists and toxicologists, delivered comprehensive care throughout the hospitalization.

Upon discharge, follow-up consultations were scheduled to monitor the patient’s recovery and assess the potential long-term effects of the overdose. These visits included clinical assessments of physical and neurological status, psychiatric evaluations, and developmental monitoring. Bloodwork tracked liver function and glucose levels, while the family received guidance on medication safety and preventive measures. A safety plan was created, and long-term follow-up appointments were arranged to evaluate any lasting effects. This coordinated and balanced approach confirmed a positive outcome, emphasizing the importance of timely intervention and ongoing support in managing the situation.

In accordance to reporting by Singh *et al.* ^7^, in cases of high olanzapine overdose it is essential to monitor serum levels of olanzapine as well, but in resource-limited setting where advanced monitoring and treatment options may be scarce, the emphasis on timely supportive care, including airway management and the prevention of secondary complications, can lead to successful outcomes even in severe cases of overdose.

## Conclusion

This case underscores the serious risk posed by olanzapine poisoning in children, particularly with high accidental doses like 180 mg consumed by our patient, the highest reported in this age group. Despite the potential for severe cardiovascular and neurological toxicity, our case demonstrated that with prompt and aggressive supportive care—including early intubation, continuous monitoring, and management of metabolic disturbances—positive outcomes are achievable even in resource-limited settings. The case also highlights the critical importance of preventive measures to avoid such accidental ingestions and the need for vigilance in managing and monitoring symptoms in pediatric patients.

## Ethical approval

Not applicable.

## Consent

Written informed consent was obtained from the patient for the publication of this case report and accompanying images. A copy of the written consent is available for review by the Editor-In-Chief of this journal on request.

## Source of funding

Not applicable.

## Author contribution

Conceptualization: P.P. Patient management: S.B., P.A., A.D., A.U., S.C. Writing—original draft: P.S.Y., P.P. Writing—review and editing: P.S.Y., P.P., S.B., P.A., A.D., A.U., S.C. Visualization and supervision: S.C. All authors have read and agreed to the final version of the manuscript.

## Conflicts of interest disclosure

The authors declare no conflicts of interest.

## Research registration unique identifying number (UIN)

Not applicable.

## Guarantor

Popular Pokhrel.

## Data availability statement

The data that support the findings of this study are available from the corresponding author upon reasonable request.

## Provenance and peer review

Not commissioned, externally peer-reviewed.
